# Molecular mechanism of tRNA binding by the *Escherichia coli* N7 guanosine methyltransferase TrmB

**DOI:** 10.1016/j.jbc.2023.104612

**Published:** 2023-03-16

**Authors:** Sarah K. Schultz, Kieran Meadows, Ute Kothe

**Affiliations:** 1 Alberta RNA Research and Training Institute (ARRTI), Department of Chemistry and Biochemistry, University of Lethbridge, Lethbridge, Alberta, Canada; 2 Department of Chemistry, University of Manitoba, Winnipeg, Manitoba, Canada

**Keywords:** RNA modification, RNA methylation, 7-methylguanosine, RNA binding protein, fluorescence, presteady-state kinetics, enzyme kinetics, transfer RNA (tRNA)

## Abstract

Among the large and diverse collection of tRNA modifications, 7-methylguanosine (m^7^G) is frequently found in the tRNA variable loop at position 46. This modification is introduced by the TrmB enzyme, which is conserved in bacteria and eukaryotes. However, the molecular determinants and the mechanism for tRNA recognition by TrmB are not well understood. Complementing the report of various phenotypes for different organisms lacking TrmB homologs, we report here hydrogen peroxide sensitivity for the *Escherichia coli ΔtrmB* knockout strain. To gain insight into the molecular mechanism of tRNA binding by *E. coli* TrmB in real time, we developed a new assay based on introducing a 4-thiouridine modification at position 8 of *in vitro* transcribed tRNA^Phe^ enabling us to fluorescently label this unmodified tRNA. Using rapid kinetic stopped-flow measurements with this fluorescent tRNA, we examined the interaction of WT and single substitution variants of TrmB with tRNA. Our results reveal the role of S-adenosylmethionine for rapid and stable tRNA binding, the rate-limiting nature of m^7^G46 catalysis for tRNA release, and the importance of residues R26, T127, and R155 across the entire surface of TrmB for tRNA binding.

tRNAs contain the greatest density and diversity of modifications among RNA species (1). Among the assortment of chemical modifications, single methylations occur at different atoms of each of the four canonical nucleobases or the ribose sugar and are introduced by a collection of unrelated methyltransferases (2, 3). Whereas some modifications are present in only certain tRNA isoacceptors in specific organisms, other modifications are highly conserved. The N7-methylguanosine (m^7^G) modification is common in bacterial and eukaryotic tRNAs and has been found in a few archaea (4). Most often, m^7^G is present at position 46 in the variable loop of tRNAs, although it has additionally been found at tRNA positions 34, 36, and 49 in a handful of organisms (4). Within tRNAs, the base at position 46 is involved in a tertiary base pair with C13-G22, where it acts as a staple connecting the variable loop and the D arm. At this position vital for tRNA tertiary structure, the m^7^G46 modification introduces a site-specific positive charge amongst the negatively charged tRNA backbone (5, 6). In *Escherichia coli*, m^7^G46 is introduced in about half of all tRNAs by TrmB, an S-adenosylhomocysteine (SAM)-dependent methyltransferase (Fig. 1) (7, 8).

**Figure 1:**
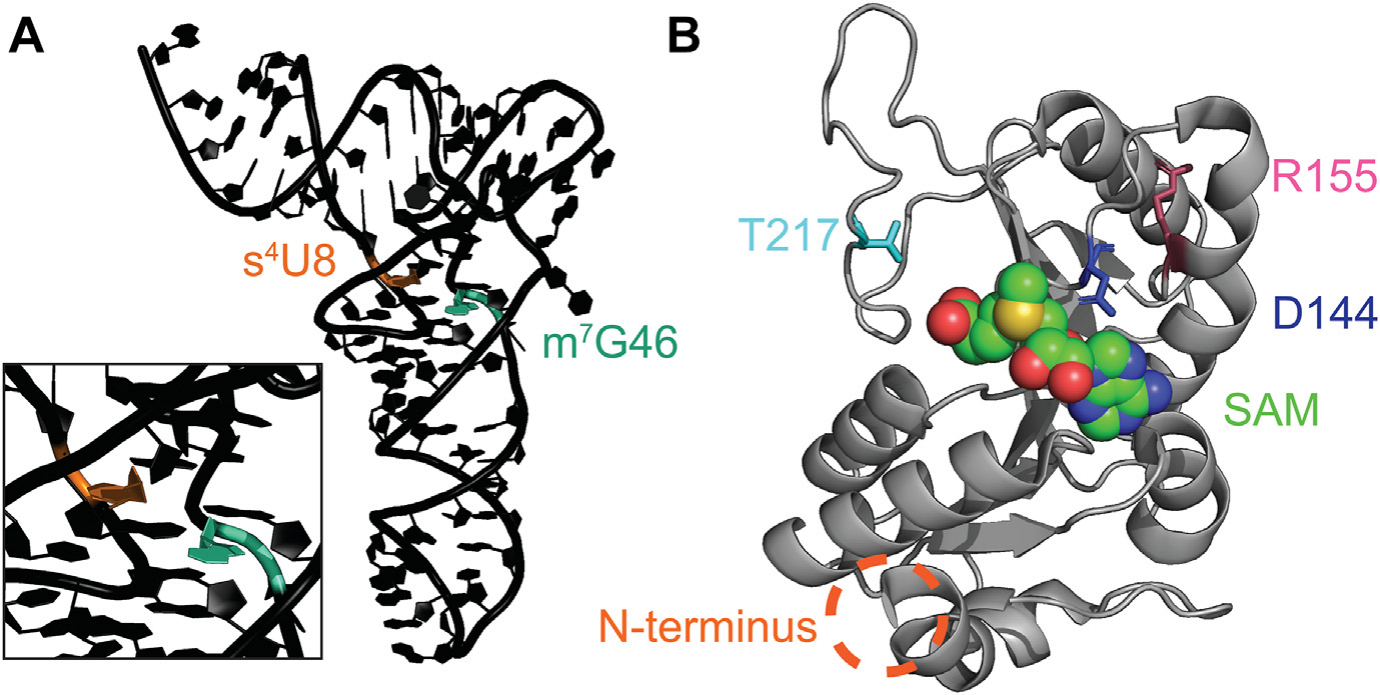
**Structures of tRNA and TrmB.***A,* crystal structure of unmodified *E. coli* tRNA^Phe^ (PDB 3L0U; (58)) highlighting the locations of G46, where TrmB introduces the 7-methylguanosine modification (m^7^G, *teal*) and U8, which was modified by ThiI and IscS to 4-thioururidine (s^4^U, *orange*) for subsequent fluorescent labeling in this study. Panel inset is a close-up view for better visualization of the U8 and G46 nucleotides. *B,* crystal structure of *E. coli* TrmB in complex with its cofactor SAM (PDB 3DXY; (8)). The methyl donor SAM is shown in *spheres* and residues addressed in this study are shown as *sticks*, with residue D144 highlighted in *blue*, T217 in *teal*, and R155 in *pink*. As the first 36 amino acids of TrmB were not resolved in this structure, the location of the N terminus, which would include residue R26, is *circled in orange.*

Similar to many tRNA modification enzymes, TrmB is nonessential in bacteria and knockout of the *E. coli trmB* gene does not impair growth under ideal conditions (7). While this observation has raised questions about the functions of TrmB and other tRNA modification enzymes, newer findings in different organisms suggest a role for the m^7^G46 modification under certain disease or stress conditions. Several recent studies have identified an importance for the human TrmB homolog METTL1 for cancer cell progression across several cancer types (reviewed in (9, 10)), wherein the m^7^G46 modification prevents degradation of specific tRNAs, leading to an increase in global translation in addition to biased translation of growth-promoting genes (11, 12, 13). Thus, this tRNA methyltransferase has gained attention as a potential biomarker for cancer prognosis and as a potential chemotherapeutic target. Moreover, human mutations resulting in the reduction of m^7^G methylation have been associated with a distinct form of microcephalic primordial dwarfism (14). Yeast lacking the TrmB homolog Trm8 display a specific growth defect in synthetic minimal media containing 2% glycerol at 38 °C (15). This phenotype is exacerbated upon deletion of certain additional tRNA modification enzymes due to rapid decay of specific tRNA isoacceptors (16). Within eubacteria, *Pseudomonas aeruginosa* lacking TrmB has been shown to be sensitive to hydrogen peroxide (H_2_O_2_) (17). A similar peroxide sensitivity has also been shown for the phytopathogenic fungus *Colletotrichum lagenarium* lacking the *trmB* homolog *aph1. C. lagenarium* cells lacking *aph1* additionally grow poorly in high-salt concentrations and are unable to infect plant cells (18). Finally, *Thermus thermophilus ΔtrmB* cells exhibit severe growth defects at high temperatures, accompanied by decreases in other tRNA modifications, tRNA stability, and protein synthesis (19).

Some structural information about TrmB and its homologs is available but we still do not know how exactly TrmB recognizes tRNA. Formation of m^7^G46 in bacteria requires only the TrmB protein; however, in eukaryotes, two nonrelated enzymes form a complex to modify tRNA. Whereas *E. coli* TrmB behaves as a monomer (7), other bacterial TrmB homologs have been shown to be homodimeric in solution, including *Bacillus subtilis, Streptococcus pneumoniae*, and *Aquifex aeolicus* TrmB homologs (8, 20, 21). In contrast, the eukaryotic TrmB homolog, known as Trm8 in yeast and METTL1 in humans, forms a heterodimer with Trm82 (yeast) or WDR4 (humans) (22, 23). Crystal structures for *E. coli*, *A. aeolicus*, *B. subtilis*, and *Saccharomyces cerevisiae* TrmB homologs have been solved alone and/or with substrate SAM or product S-adenosylhomocysteine (SAH) (8, 20, 23, 24, 25). These structures have revealed that all TrmB homologs are class I methyltransferases deviating from the classic Rossman-fold only by an insertion between two β-strands (8, 20). In *E. coli* TrmB, this insertion lacks secondary structure (8), whereas an α-helix is evident in other TrmB/Trm8 structures (20, 23). A structure for bacterial TrmB in complex with tRNA does not yet exist and as such, we are lacking an understanding of how TrmB binds its substrate tRNA.

A prior study of *E. coli* TrmB identified several TrmB residues necessary for TrmB methylation activity *in vitro* but did not distinguish which of these residues are directly responsible for catalysis and which are involved in tRNA binding (26). Single-residue alanine substitutions at conserved residues R26, D144, H151, R154, R155, D180, T217, and E220 reduced multiple-turnover methylation activity to less than 10 % of that of the WT enzyme, with substitutions at D144, R154, and R155 abolishing methyltransferase activity completely under these conditions (26). Steady-state kinetic analysis for several equivalent single alanine substitutions in *A. aeolicus* TrmB has suggested that TrmB D144A, R155A, and T217A variants display slower methylation than WT TrmB, whereas SAM binding is unaffected for these variants (27). The authors of this study proposed a potential catalytic mechanism wherein D180 and the adjacent T179 residue (*E. coli* TrmB numbering) interact with the N1-proton and O6 atom of the target guanosine 46 base, respectively, thus positioning the N7 atom for nucleophilic attack by the SAM methyl group (27).

Here, we investigate the function of TrmB for *E. coli* fitness and dissect its interaction with tRNA using a novel assay. Thereby, we report that the model bacterium *E. coli* is sensitive to hydrogen peroxide stress in the absence of TrmB. In order to gain a better understanding as to how this tRNA modification enzyme binds tRNA, we prepared partially modified tRNA^Phe^ containing the 4-thiouridine 8 (s^4^U8) modification using purified ThiI (also known as TtuI (28, 29)) and IscS enzymes. The reactivity of the s^4^U8 modification was then used to fluorescently label the tRNA for presteady-state kinetic analysis using a stopped-flow instrument. Moreover, to investigate the roles of specific TrmB residues for tRNA binding, we analyzed four TrmB variants: R26A, D144A, R155A, and T217A. The binding of these variant enzymes to tRNA was examined in real time using our stopped-flow assay, the affinity of these enzymes for tRNA was determined using nitrocellulose filtration, and the methylation activity of TrmB WT and variants was assessed in single-turnover assays. Taken together, our results suggest that tRNA binding by TrmB is a complex, multistep process aided by prior binding of SAM.

## Results

### *E. coli ΔtrmB* grows slowly in the presence of hydrogen peroxide

**Figure 2:**
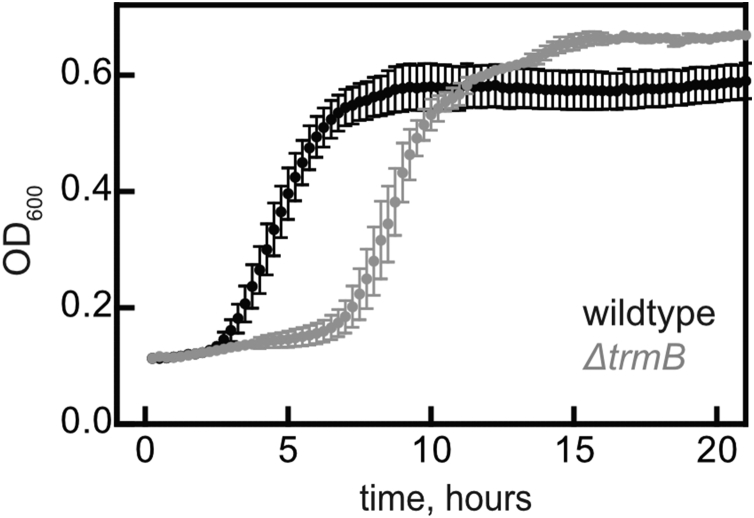
***E. coli* BW25113 *ΔtrmB* grows slowly in LB medium-containing hydrogen peroxide.** Four biological replicates for WT *E. coli* BW25113 (*black*) and *E. coli* BW25113 *ΔtrmB* (*gray*) were seeded with a starting A_600_ of 0.1 in a 96-well plate. Average A_600_ values recorded every 15 min and error bars represent SEM.

*E. coli trmB* is nonessential for cell growth and no growth phenotypes have been reported for the *E. coli ΔtrmB* strain under ideal growth conditions (7). Since previous work has shown *P. aeruginosa* and the parasitic fungus *C. lagenarium* lacking the *trmB* gene grow slowly in media containing hydrogen peroxide, we asked whether *E. coli* lacking *trmB* displays the same phenotype (17, 18). Indeed, *E. coli* BW25113 *ΔtrmB* reproducibly grows slower than its parental strain in LB medium supplemented with H_2_O_2_. Although the exponential and stationary phases are not affected by *trmB* KO, the lag phase for the *ΔtrmB* cells is longer than that of the WT (Fig. 2). Thus, an increased sensitivity to hydrogen peroxide in the absence of *trmB* seems to be common between *E. coli*, *P. aeruginosa*, and the eukaryotic fungus *C. lagenarium*.

### Partial modification and fluorescent labeling of tRNA^Phe^ for rapid kinetic analysis of tRNA binding by TrmB

**Figure 3:**
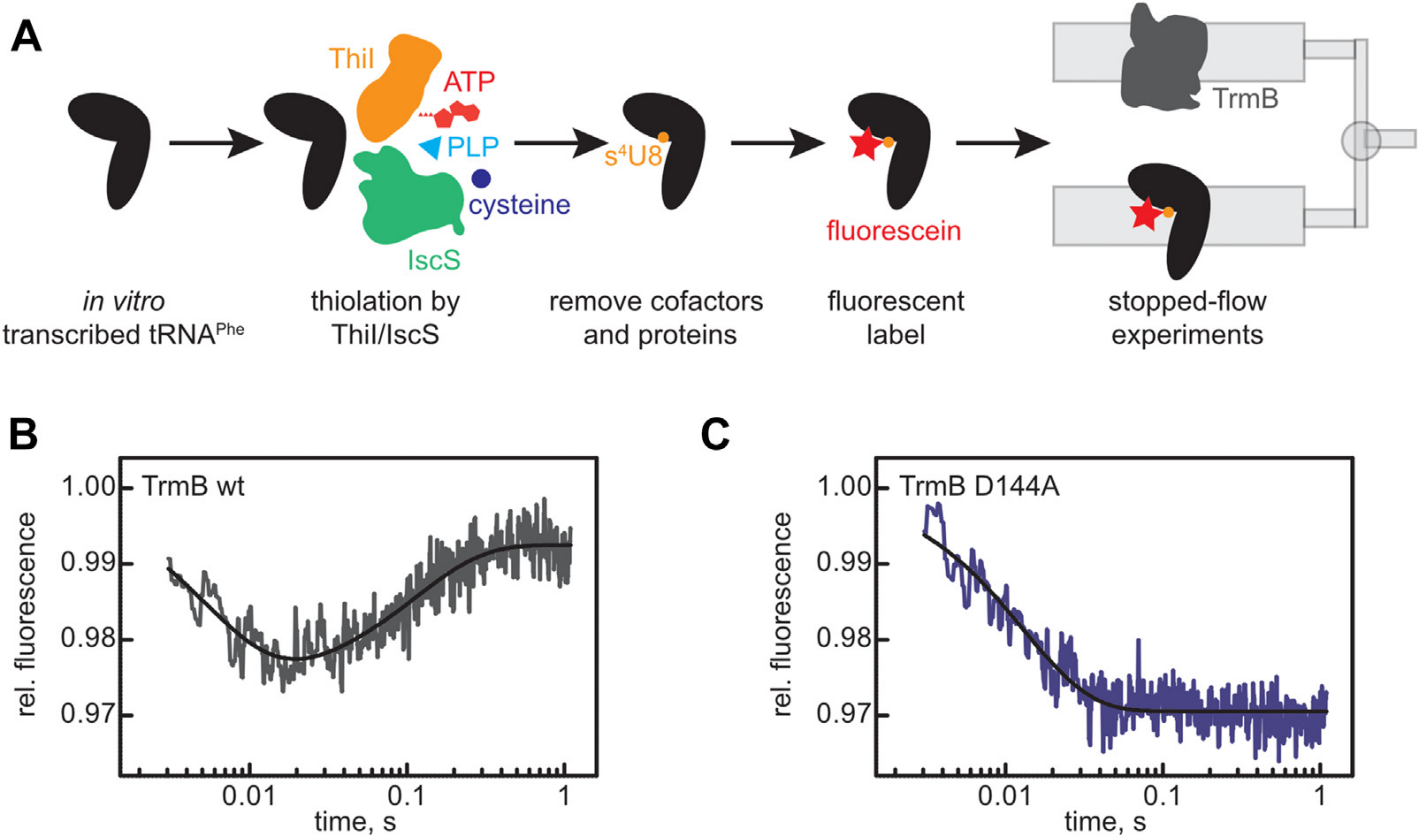
**Partial modification and fluorescent labeling of tRNA**^**Phe**^**for rapid kinetic stopped-flow experiments.***A,* labeling scheme detailing the introduction of s^4^U8 by ThiI and IscS, modified tRNA purification, and fluorescent labeling with fluorescein for use in rapid kinetic assays. *B,* rapid mixing of 15 μM WT TrmB and 50 μM SAM with 1 μM fluorescein-s^4^U8-tRNA^Phe^. The data was fit to a two-exponential equation to determine apparent rate constants: *k*_app1_: 180 s^−1^ and *k*_app2_: 9 s^−1^. *C*, rapid mixing of catalytically inactive 15 μM TrmB D144A and 50 μM SAM with 1 μM fluorescein-s^4^U8-tRNA^Phe^. Fitting the data with a one-exponential equation determined a *k*_app1_ of 77 s^−1^.

In order to examine how TrmB is binding tRNA, we sought to introduce a fluorescent label into tRNA that would enable a fluorescence change that could be observed in real time using stopped-flow fluorescence spectrometry. Within the three-dimensional tRNA structure, the s^4^U8 residue within the acceptor stem is in close proximity to the TrmB target nucleoside G46 (Fig. 1*A*). Previous studies of tRNAs interacting with the ribosome have exploited the reactive nature of the s^4^U8 thiol modification to introduce different fluorescent dyes attached to iodoacetamide into native tRNA isoacceptors purified from cells (30). In order to study the interaction of TrmB with its substrate tRNA lacking m^7^G46 and other modifications, we instead *in vitro* transcribed tRNA and introduced the single s^4^U8 modification using purified ThiI and IscS enzymes for fluorescent labeling with 5-iodoacetamidofluorescein (5-IAF) (Fig. 3*A*). Single-turnover methylation assays confirm that fluorescein-s^4^U-tRNA^Phe^ can be rapidly methylated (Fig. S1*A*).

Using the fluorescein-s^4^U8-tRNA^Phe^, we monitored the binding of WT TrmB to tRNA in the millisecond-to-seconds range using a stopped-flow apparatus. In the presence of 50 μM SAM, mixing 15 μM TrmB with 1 μM fluorescein-s^4^U8-tRNA^Phe^ resulted in a biphasic signal with an initial fluorescence decrease followed by a subsequent fluorescence increase (Fig. 3*B*). This curve was fit with a 2-exponential equation, yielding apparent rates of 180 s^−1^ (*k*_app1_) and 9 s^−1^ (*k*_app2_) (Fig. 3*B*). In contrast, mixing fluorescein-s^4^U8-tRNA^Phe^ with the active site variant TrmB D144A resulted in a single fluorescence decrease with an apparent rate of 77 s^−1^ (Fig. 3*C*). As we will show below, this variant is deficient only in methylation but not tRNA binding. In the presence of SAM, WT TrmB forms product (26, 31). Thus, the fluorescence decrease reflects an event related to tRNA binding prior to methylation. Conversely, the fluorescence increase, that we observed only with the WT enzyme, is likely to occur at a step after catalysis and may reflect release of the methylated tRNA.

### TrmB variant binding and methylating tRNA^Phe^

**Figure 4:**
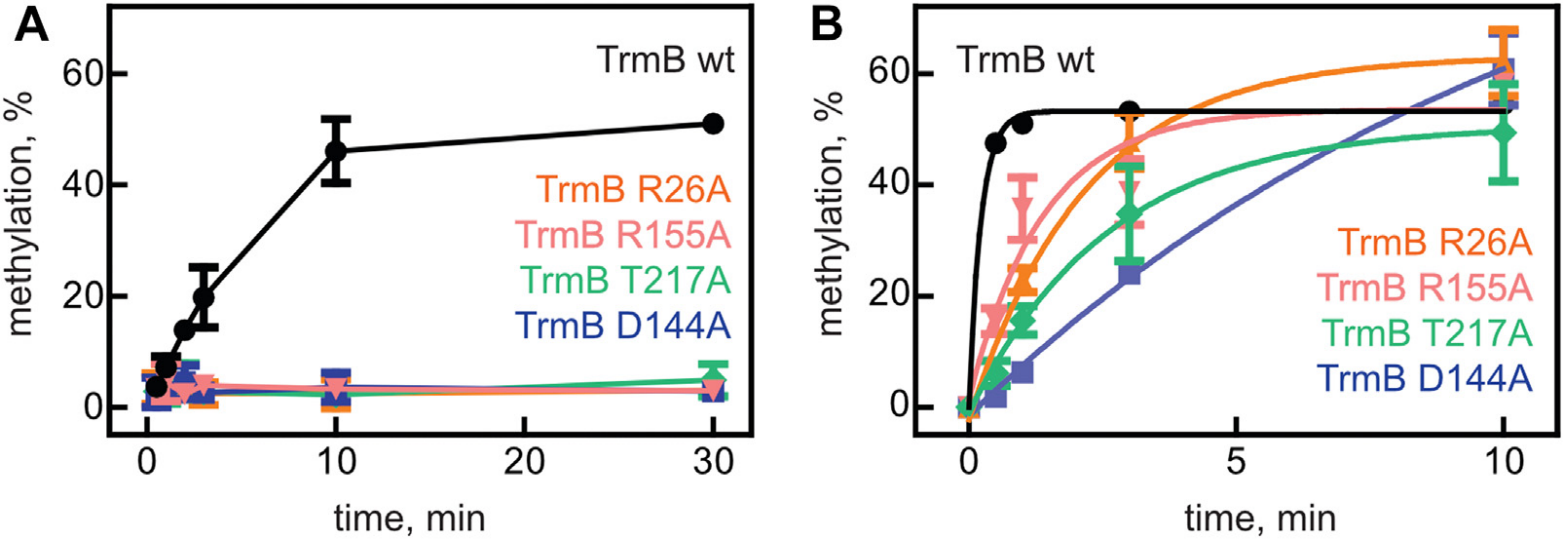
**Time courses for methylation of tRNA**^**Phe**^**by TrmB WT and variants.***A,* methylation of 1 μM tRNA^Phe^ under multiple turnover conditions using 50 nM TrmB variants and (*B*) methylation under single turnover conditions using 5 μM TrmB variants, both in the presence of 50 μM tritium-labeled SAM. Percent methylation was plotted against time. For the single-turnover methylation experiments, apparent rates of methylation for each TrmB variant were determined by fitting with a single exponential equation and rates are reported in Table 1. Note that methylation by TrmB WT shown here acts as visual comparison for variant TrmB methylation; the rate of TrmB WT methylation was previously determined using rapid kinetic quench flow (31).

As previous work with *E. coli* TrmB variants has measured only overall tRNA methylation activity, but not tRNA binding, we investigated which TrmB residues are important for tRNA binding. To this end, we prepared two TrmB variants that were previously shown to be less than 10% as active in multiple-turnover methylation assays compared to WT TrmB (26) that we hypothesized would retain tRNA binding ability while being unable to methylate tRNA: TrmB D144A and T217A (Fig. 1*B*). Here, we confirm that both residues are indeed vital for multiple-turnover methylation by TrmB (Fig. 4*A*). However, we find that at high TrmB D144A or TrmB T217A concentrations, methylation does slowly occur and reaches a similar final methylation end level as WT TrmB, with the apparent rate of methylation >150-fold slower for TrmB D144A and 35-fold slower for TrmB T217A than for WT TrmB (Fig. 4*B* and Table 1). Both residues are proposed to participate in methylation, wherein the side chain of the aspartate equivalent to D144 has been shown to form hydrogen bonds with N6 of the SAM adenosine moiety in *B. subtilis* TrmB (25). Residue T217 is present within the TrmB-specific insertion that is unstructured in *E. coli* TrmB (8). Within *B. subtilis* TrmB, this threonine residue is located at the bottom of the SAM-binding pocket, wherein its side-chain hydrogen bonds to the SAM methionyl moiety (20, 25). However, this interaction is not observed within the *E. coli* TrmB–SAM complex (8) and thus T217 may only move in close enough proximity to interact with SAM upon structural rearrangement after tRNA binding. Alternatively, this residue may play a different role in *E. coli* TrmB.

**Table 1:** Average apparent methylation rates for TrmB variants at 37 °C

TrmB variant	*kapp,* min^−1^
TrmB WT	14 ± 2^a^
TrmB D144A	0.09 ± 0.04
TrmB T217A	0.4 ± 0.1
TrmB R26A	0.5 ± 0.1
TrmB R155A	0.7 ± 0.2

a Apparent rate for TrmB WT at 37 °C was previously determined using rapid kinetic quench flow (31).

**Figure 5:**
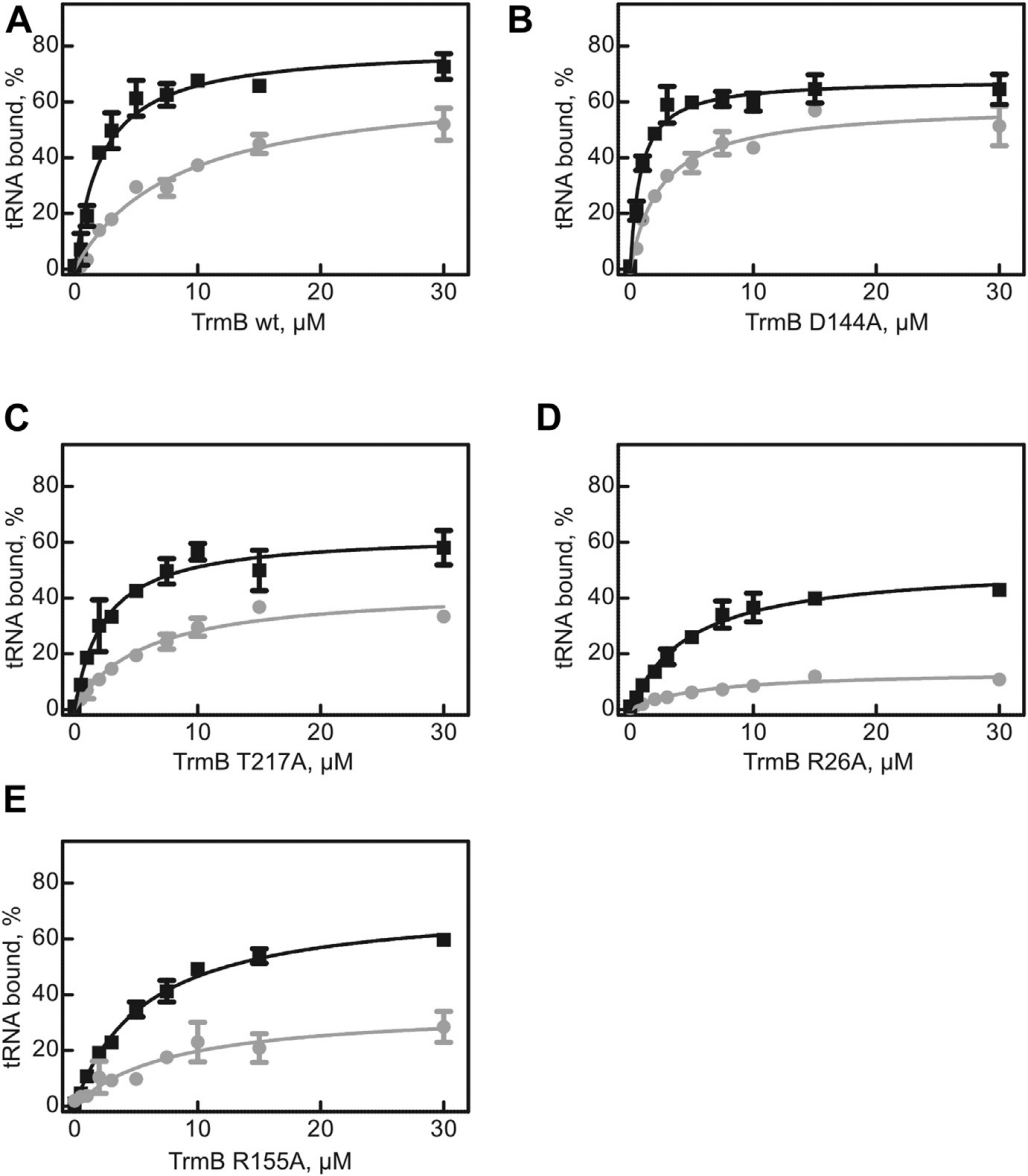
**tRNA binding by TrmB WT and variants.** To determine the affinity of TrmB in the presence (*black squares*) or absence (*gray circles*) of 50 μM SAM, 20 nM of [^3^H]tRNA^Phe^ was incubated for 10 min with increasing concentrations of TrmB WT (*A*), TrmB D144A (*B*), TrmB T217A (*C*), TrmB R26A (*D*), or TrmB R155A (*E*). Percent tRNA bound was determined by nitrocellulose filtration. Averages of at least three experiments are shown with error bars representing SD. The data was fit with a hyperbolic equation to determine the dissociation constants (see Table 2).

To measure the affinity of each TrmB variant for tRNA^Phe^, nitrocellulose filter binding using tritium-labeled tRNA was performed. Previously, we have shown that WT TrmB in the absence of SAM has a relatively low affinity for tRNA with a dissociation constant of 6.6 μM (Fig. 5*A* and Table 2) (31). When TrmB WT is preincubated with SAM, the affinity of TrmB for tRNA increases about 3-fold to 2.1 μM (Fig. 5*A* and Table 2). Thus, prior binding of SAM to TrmB has a positive allosteric effect on subsequent tRNA binding, as has previously been shown for TrmA (32).

**Table 2:** Average dissociation constants (K_D_) for TrmB WT and variants binding to [^3^H]tRNA^Phe^ in the presence and absence of 50 μM SAM

TrmB variant	+SAM, μM^a^	−SAM, μM
TrmB WT	2.1 ± 0.5	6.6 ± 1.9
TrmB D144A	0.8 ± 0.2	2.5 ± 0.7
TrmB T217A	2.6 ± 0.6	5.4 ± 1.4
TrmB R26A	5.1 ± 0.8	6.4 ± 2.5
TrmB R155A	5.8 ± 0.9	10 ± 2.8

a TrmB binding to tRNA in the presence of SAM for 10 min results in m^7^G46 formation such that the affinity of TrmB to its product rather than its substrate is measured.

Next, we examined the binding of TrmB D144A and T217A variants to tRNA^Phe^. In both the absence and presence of SAM, TrmB D144A binds tRNA with a 2.5-fold higher affinity than the WT enzyme, with a *K*_D_ of 2.5 μM and 0.8 μM, respectively (Fig. 5*B* and Table 2). Additionally, we observed that alanine substitution at residue T217 does not change the tRNA affinity in the presence and absence of SAM compared to TrmB WT (Fig. 5*C* and Table 2).

Moreover, we prepared two additional TrmB variants, that were previously shown to be less than 10% as active as WT TrmB in multiple turnover assays and we hypothesized that these TrmB variants would be deficient in tRNA binding: R26A and R155A (Fig. 1*B*). Residue R26 is present within the N-terminal extension of TrmB that is presumably flexible because this region was not resolved in the *E. coli* TrmB structure (8). As a positively charged residue, R26 may contribute to tRNA binding *via* electrostatic interactions. Another positively charged residue, R155, is one of two adjacent conserved arginine residues along with R154. Previous work showed that alanine substitution of either residue abolishes methylation activity under multiple-turnover conditions (26). Similarly, we find both variants are not active in multiple-turnover conditions (Fig. 4*A*), but slowly methylate tRNA in single-turnover conditions, each displaying an apparent rate that is ∼30-fold slower than WT TrmB (Fig. 4*B* and Table 1).

In the absence of SAM, TrmB R26A has a similar, low affinity for tRNA as TrmB WT but in the presence of SAM its tRNA affinity is 2.5-fold lower (Fig. 5*D* and Table 2). Notably, the maximum binding amplitude for TrmB R26A in the absence of SAM is very low, with only around 20% tRNA binding, further suggesting that this TrmB variant is deficient in stable tRNA binding (Fig. 5*D*). In the absence of SAM, TrmB R155A binds tRNA with a *K*_D_ of 10 μM, which is lower than the affinity of TrmB WT for tRNA (Fig. 5*E* and Table 2). Similarly, in the presence of SAM, the affinity of TrmB R155A is 3-fold lower than the TrmB WT (Fig. 5*E* and Table 2). Like the R26A variant, the maximal level of tRNA bound by TrmB R155A is low, reaching only about 30% tRNA bound (Fig. 5*E*). Thus, substitution at residues R26 and R155 negatively affect tRNA binding.

### Rapid kinetic analysis of TrmB variants

**Figure 6:**
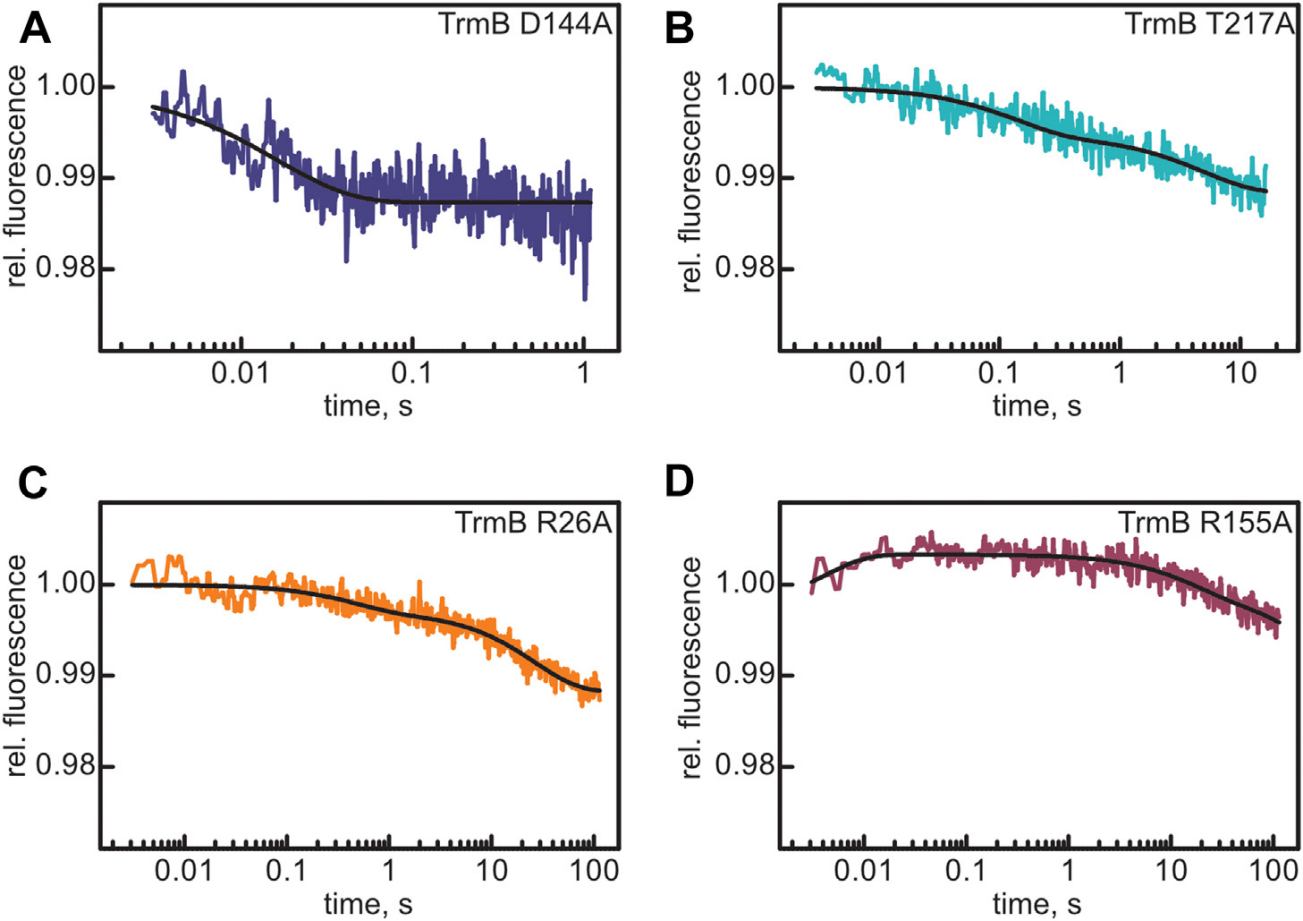
**Rapid kinetic analysis of TrmB variant binding to tRNA.** Time courses displaying rapid mixing of 15 μM TrmB variants with 1 μM fluorescein-s^4^U8-tRNA^Phe^ in a stopped-flow apparatus. Each trace is an average of at least eight independent time courses. Note the different x-axis range between panels. The time course for TrmB D144A (*A*) was fit to a one-exponential equation with a *k*_app1_ of 62 ± 5 s^−1^, whereas data for TrmB T217A (*B*) was fit to a two-exponential equation with a *k*_app1_ of 8 ± 1 s^−1^ and a *k*_app2_ of ∼0.2 s^−1^. Time courses for TrmB R26A (*C*) were fit to a two-exponential equation, with a *k*_app1_ of 2 ± 0.2 s^−1^ and *k*_app2_ of ∼0.04 s^−1^. Finally, data for TrmB R155A (*D*) was fit to a three-exponetial equation, revealing a *k*_app1_ of 176 ± 46 s^−1^, *k*_app2_ of 2 ± 1 s^−1^, and a *k*_app3_ of ∼0.03 s^−1^. Apparent rates are summarized in Table S1.

To monitor the interaction between TrmB variants and tRNA in real time, we repeated the stopped-flow assays using fluorescein-s^4^U-tRNA^Phe^ with TrmB variants. Importantly, for all TrmB variants, no significant product formation occurs even at high TrmB concentrations during the course of the stopped-flow experiments (1–100 s; Fig. 4*B*). Binding of the TrmB D144A variant to tRNA in the absence of SAM (Fig. 6*A*) displays a similar single-phase fluorescence decrease as seen when mixing this variant and fluorescein-s^4^U8-tRNA^Phe^ in the presence of SAM (Fig. 3*B*). Fitting the binding curve of TrmB D144A in the absence of SAM with an exponential equation yielded an apparent rate of about 60 s^−1^ which is comparable to the apparent rate of 77 s^−1^ observed in the presence of SAM.

The affinity of the TrmB T217A variant is similar to that of the WT enzyme but reduced compared to TrmB D144A (Fig. 5 and Table 2). Notably, the fluorescence change observed upon mixing TrmB T217A with fluorescein-s^4^U8-tRNA^Phe^ is different from TrmB WT and D144A. Here, we observe two subsequent fluorescence decreases, with apparent rates of 8 s^−1^ (*k*_app1_) and 0.2 s^−1^ (*k*_app2_) (Fig. 6*B*). Likewise, mixing TrmB R26A with fluorescein-s^4^U8-tRNA^Phe^ reveals two fluorescence decreases with a *k*_app1_ of 2 s^−1^ and *k*_app2_ of 0.03 s^−1^ (Fig. 6*C*). Finally, the binding of TrmB R155A with fluorescein-s^4^U8-tRNA^Phe^ in real time occurs in three phases with an initial fluorescence increase (*k*_app(increase)_) with a rate of 176 s^−1^, followed by two decreases in fluorescence with rates of 3 s^−1^ (*k*_app1_) and 0.03 s^−1^ (*k*_app2_) (Fig. 6*D*). Thus, our rapid kinetic assays reveal fast tRNA binding only in the case of the WT enzyme and the catalytically impaired variant TrmB D144A. In accordance with the defects in tRNA binding for TrmB T217A, R26A, and R155A compared to the TrmB variant D144A observed in equilibrium binding conditions in the nitrocellulose-filtration assays (Fig. 5 and Table 2), the TrmB T217A, R26A, and R155A variants display vastly different tRNA-binding time courses, suggesting that substitutions to these residues significantly perturb how these variants bind tRNA. (Fig. 6 and Table S1).

### Rapid kinetic analysis of TrmB binding and methylating tRNA^Phe^

**Figure 7:**
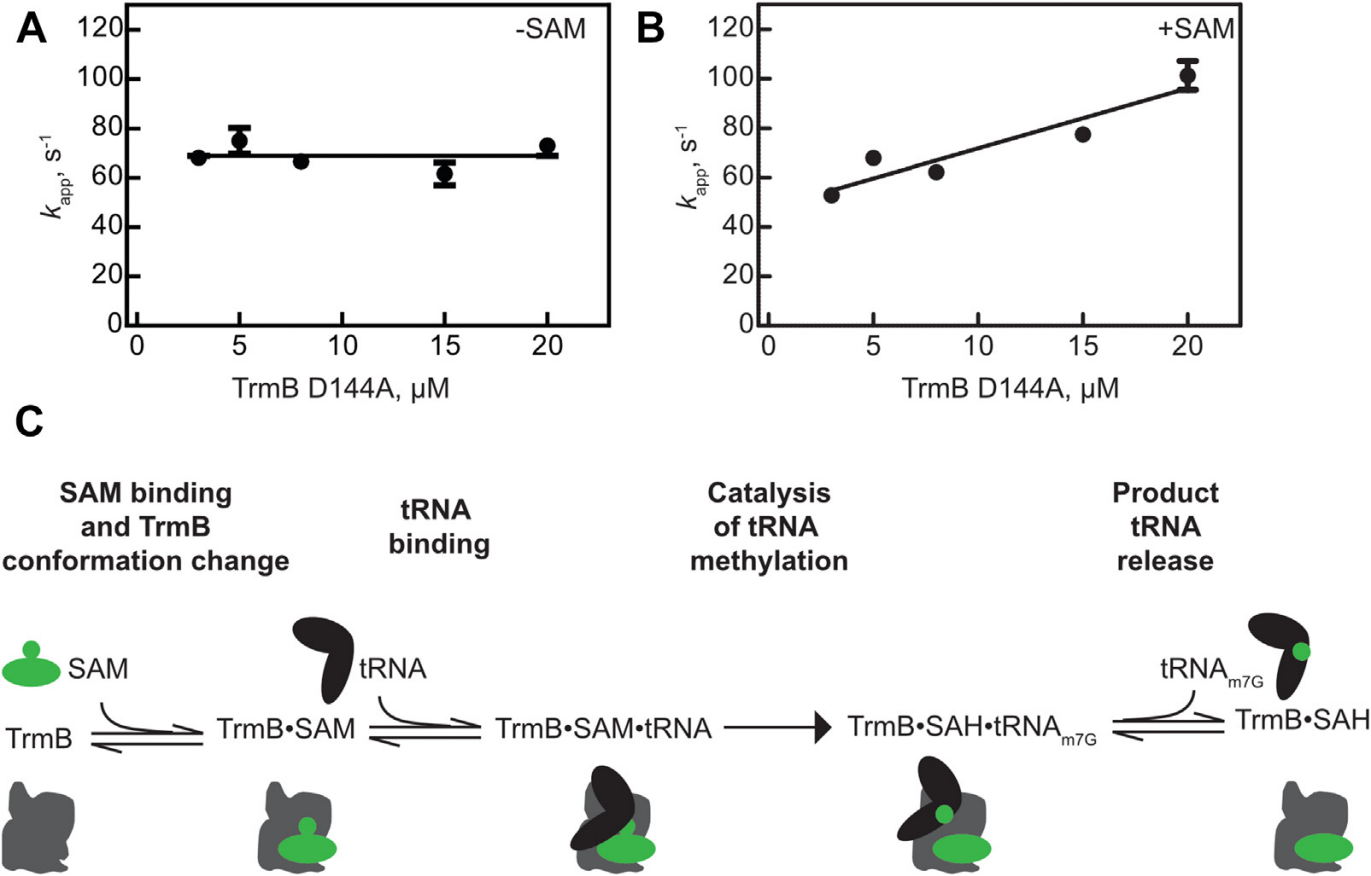
**Concentration dependence of tRNA binding by TrmB D144A.** Time courses of TrmB D144A binding to 1 μM fluorescein-s^4^U8-tRNA^Phe^ were fit to single exponential equations to determine the concentration dependence of apparent rates. In the absence of SAM (*A*), the *k*_app_ is not concentration-dependent and the horizontal line represents the average apparent rate of 69 ± 5 s^−1^. In the presence of SAM (*B*), the apparent rate of tRNA binding by TrmB is dependent on TrmB concentration. Thus, data was fit with a linear regression to determine the slope representing the association rate constant (*k*_1_) of 2.4 ± 0.5 μM^−1^ s^−1^. *C,* Kinetic mechanism for tRNA methylation by TrmB. Herein, SAM binding to TrmB prompts a conformational change within TrmB, which facilitates rapid and stable tRNA binding by TrmB. tRNA methylation occurs slowly and is followed by the rapid and reversible release of product (methylated) tRNA.

In order to kinetically characterize the association of TrmB to tRNA^Phe^ in the absence of methylation, we used the TrmB D144A variant that is highly deficient in catalysis but not tRNA binding (Figs. 5 and 6, (26)). Increasing concentrations of TrmB D144A were rapidly mixed with fluorescein-s^4^U8-tRNA^Phe^ and one-exponential equations were fit to the resulting curves. In the absence of the methyl donor SAM, the apparent rate is not dependent on enzyme concentration, with an average rate of 69 ± 5 s^−1^ (Fig. 7*A*). This suggests that in the absence of SAM the observed apparent rate is indicative of a unimolecular conformational change, rather than a bimolecular binding event. In contrast, when TrmB D144A is prebound to SAM, the apparent rate of tRNA binding is concentration-dependent and therefore is indicative of a bimolecular interaction. Determination of the slope reveals an association rate constant, *k*_1_, of 2.4 ± 0.5 μM^−1^ s^−1^ (Fig. 7*B*). As we will further discuss below, these results can be explained if a SAM-induced conformational change in TrmB is required for stable binding of tRNA.

To complement the kinetic analysis of tRNA binding to TrmB, we also kinetically characterized the methylation activity of TrmB WT using single-turnover quench-flow experiments and [^3^H]SAM (Fig. S1). At 20 °C, the apparent rate for tRNA methylation is 0.04 s^−1^ and this apparent rate is not concentration dependent (Fig. S1*B*). This observation suggests that methylation is not limited by tRNA binding.

## Discussion

### Peroxide sensitivity of *E. coli ΔtrmB*

In this study, we report for the first time a growth phenotype for an *E. coli ΔtrmB* strain, wherein *ΔtrmB* cells grow slowly in hydrogen peroxide-containing LB medium. This phenotype matches previous reports of phenotypes for *trmB* KOs in *P. aeruginosa* and *C. lagenarium* (17, 18). During the course of our study, this phenotype was reported to be absent for the *E. coli* BW25113 *ΔtrmB* strain, even in the presence of 10 mM H_2_O_2_ (33). However, this phenotype is highly reproducible in our experiments (Fig. 2) and even the BW25113 WT strain does not significantly grow in the presence of 10 mM H_2_O_2_ within the small volume of 96-well plates (data not shown). This lack of growth for the BW25113 strain at high H_2_O_2_ concentrations has recently been corroborated by others (34). Given the unstable nature of hydrogen peroxide rendering it difficult to use precise concentrations and the conserved nature of this phenotype across different organisms, we conclude that TrmB is important for fitness during oxidative stress.

It is notable that sensitivity to hydrogen peroxide stress is present in two different bacterial species, in addition to *C. lagenarium*, a eukaryote. This suggests the m^7^G46 modification and/or TrmB plays a conserved role in mediating oxidative stress. Within *P. aeruginosa*, TrmB was found to mediate the peroxide stress response by optimizing translation of Phe and Asp codons, which are enriched within the *P. aeruginosa* catalase genes (17). Interestingly, *E. coli* catalase genes *katE* and *katG* similarly display an overabundance of Phe(UUC), Asp(GAC), and Asp(GAT) codons. The precise mechanism how TrmB enhances Phe/Asp translation remains unclear. It could be that the m^7^G46 modification is directly important for tRNA aminoacylation or for the role of tRNA on the ribosome during translation or m^7^G46 could act to increase the cellular stability of certain tRNAs, thereby increasing their cellular abundances and availability for translation. Considering the demonstrated importance of the m^7^G46 modification by Trm8/Trm82 for preventing rapid decay of tRNA^Val^_(AAC)_ in yeast (16), in addition to the recent evidence that m^7^G46 formation by METTL1/WDR4 in mammals is important for the stability of at least tRNA^Arg^_(UCU)_ (11) or tRNA^Lys^_(CUU)_ (35) and tRNA^Lys^_(UUU)_ (13), it is likely that methylation by TrmB is important for the cellular stability of certain tRNAs in bacterial cells. This possibility will be interesting to examine in the future. Moreover, given the specific importance of m^7^G46 for only certain tRNAs across different organisms, despite the fact that TrmB modifies almost all tRNAs containing short variable loops, investigation of the mechanism underlying why TrmB is important for peroxide tolerance in *E. coli* and whether or not this is similar to the determined mechanism for *P. aeruginosa* is interesting to consider.

### *In vitro* partial modification and fluorescent tRNA labeling

Fluorescent labeling of tRNAs at different specific internal sites by taking advantage of the reactivity of native tRNA modifications has been used to study the dynamics of tRNAs on the ribosome during protein translation (30, 36). However, few studies have prepared *in vitro* transcribed tRNAs with only a select modification for internal fluorescent modification of a mostly unmodified tRNA (37, 38). Using *in vitro* transcribed tRNA is advantageous for studying its interaction of tRNA-modifying enzymes because this allows for examination of the binding of a modification enzyme to its substrate (rather than product), unmodified tRNA, without the laborious process of extracting specific tRNA isoacceptors from an appropriate KO strain. Moreover, previous work has suggested TrmB homologs tend to modify tRNA in the relatively early stages of tRNA maturation (39, 40) and *E. coli* TrmB seems to not have a preference for tRNA modification status (31). This suggests that a mostly unmodified tRNA is an appropriate substrate for studying the interaction between TrmB and tRNA. As ThiI has few sequence constraints for modifying U8-containing tRNAs and instead recognizes the overall L-shaped structure (41), this strategy can be used to introduce s^4^U8 within a wide selection of tRNAs. Thus, the here described preparation of fluorescently labeled, partially modified tRNA for kinetics studies lays the foundation for study of additional tRNA modification enzymes or other tRNA-interacting enzymes, demonstrating another use for *in vitro*-modified tRNAs (42).

### Molecular mechanism of *E. coli* TrmB interacting with tRNA

As we have previously shown, in comparison to other tRNA modification enzymes, *E. coli* TrmB has a relatively low affinity for tRNA with a *K*_D_ of about 6 μM in the absence of its cofactor SAM (Fig. 5 and Table 2) (31). Interestingly, *E. coli* TrmB binds tRNA with a lower affinity than its characterized homolog; homodimeric *B. subtilis* TrmB was shown to bind tRNA^Phe^ in the absence of SAM with an affinity of about 0.1 μM using fluorescence anisotropy (25). This raises the possibility that TrmB enzymes with different quaternary structures may have distinct tRNA binding mechanisms. As such, the affinity may increase for homodimeric enzymes compared to monomeric *E. coli* TrmB. No investigations concerning the affinity of the yeast Trm8/Trm82 heterodimer for tRNA have been conducted to date, and it is possible the heterodimeric complex again binds tRNA differently compared to monomeric bacterial TrmB. Supporting this possibility, differences in tRNA structural requirements have been identified between homodimeric *A. aeolicus* TrmB and heterodimeric yeast Trm8, wherein both enzymes require the T stem for modification activity but only yeast Trm8 additionally requires the D stem (43, 44). Moreover, the R26 residue, that we demonstrate here to be important for tRNA binding (Table 2, Figs. 5 and 6), is not conserved in *B. subtilis, S. cerevisiae* or human TrmB homologs, and the N-terminal region present in mesophilic TrmB proteins is entirely absent in *A. aeolicus* TrmB. Notably, TrmB is one of several tRNA modification enzymes that utilizes only a single subunit in bacteria but requires an auxiliary protein for catalysis in eukaryotes (45, 46). As such, a similar difference in tRNA affinity may exist for other bacterial tRNA modification enzymes and their eukaryotic two-subunit homologs.

Interestingly, we are demonstrating here that the affinity of TrmB WT and variants for tRNA is significantly increased in the presence of SAM (Table 2). Thus, prior binding of the cofactor SAM has a positive allosteric effect on the interaction of TrmB with tRNA, presumably by ordering and preorienting the active site. Previously, we have described a similar positive allostery for SAM and tRNA binding for the methyltransferase TrmA responsible for the m^5^U54 modification in tRNAs (32). Alternatively, the presence of SAM may play a more direct role in tRNA binding by contributing interactions with the tRNA itself. Recent structures of the human TrmB homolog METTL1 in complex with its partner protein WDR4, tRNA and SAH support the hypothesis that prebinding of SAM/SAH by TrmB preorients the TrmB active site (47, 48); however, as the quaternary structures of *E. coli* TrmB and human METTL1 are different, a ternary structure of TrmB in complex with SAM/SAH and tRNA would be required to fully explain the allosteric role of SAM in tRNA binding by TrmB.

In order to characterize the molecular mechanism of TrmB’s interactions with tRNA, we report here a novel fluorescent assay enabling rapid kinetic studies of tRNA binding to TrmB (Fig. 3*A*). In the stopped-flow assay, we observe how fast TrmB binds to unmodified substrate tRNA giving us insight into the initial steps of TrmB’s molecular mechanism. Thus, the stopped-flow assay provides different types of information (kinetics of substrate binding) about TrmB than the nitrocellulose-filtration assay (affinity of product binding). By comparing how tRNA interacts with WT TrmB compared to catalytically impaired TrmB D144A, we can gain insight into the kinetic mechanism of tRNA binding for this tRNA modifying enzyme. In these rapid kinetic stopped-flow experiments, we observe first a fluorescence decrease when either WT TrmB or TrmB D144A interact with fluorescein-s^4^U8-tRNA in the presence of SAM. This fluorescence decrease upon tRNA binding may reflect partial unfolding of the tRNA structure as TrmB accesses its target base, G46, which is buried within the tRNA elbow forming a base pair triple with C13-G22. Notably, the apparent rate for the fluorescence decrease upon tRNA binding in the presence of SAM is 2-fold higher for WT TrmB than TrmB D144A (Fig. 3), suggesting that substitution of D144 for alanine slightly slows the association of TrmB with unmodified tRNA relative to WT TrmB. This is supported by the fact tRNA methylation by TrmB D144A appears to be partially limited by tRNA binding (Fig. 4) as some methylation activity is observed in single-turnover methylation assays using 5 μM TrmB concentrations but not in multiple-turnover methylation assays using 50 nM TrmB.

Following the first fluorescence decrease in the rapid kinetic stopped-flow assays with WT TrmB in the presence of SAM discussed above, we then observe a second fluorescence increase with an apparent rate of ∼9 s^−1^. Presumably, this phase reflects the release of the modified tRNA, as this fluorescence change is absent when the catalytically impaired D144A variant is mixed with tRNA in the presence of SAM (Fig. 3*C*). We have previously reported the *k*_app_ for tRNA methylation is 0.2 s^−1^ at 37 °C (Table 1, (31)) and measured here that the apparent rate for tRNA methylation at 20 °C is 0.04 s^−1^ and is not concentration-dependent (Fig. S1*B*), suggesting that methylation is not limited by tRNA binding. Thus, catalysis of m^7^G46 formation may be the rate-limiting step in the molecular mechanism of TrmB which is followed by rapid product tRNA release (Fig. 7*C*). The apparent rate of the fluorescence increase observed with TrmB WT presumably reflects a combination of slow tRNA release and some product tRNA rebinding as the affinity of TrmB WT for modified tRNA is 2.1 μM (Table 2). Notably, we have likewise reported that many tRNA pseudouridine synthases are characterized by a rate-limiting catalytic step (49, 50).

To gain further insight into the mechanism of tRNA binding by TrmB, we performed a titration of TrmB D144A binding to tRNA in the stopped-flow experiments (Fig. 7). In the absence of SAM, we observed that the apparent rate, *k*_app_, is not dependent on enzyme concentration, suggesting a unimolecular conformational change rather than a bimolecular binding event. In contrast, *k*_app_ was found to be dependent on TrmB D144A concentration in the presence of SAM, which suggests that in this case we observe a bimolecular binding event. This different kinetic behavior in the presence and absence of SAM indicates that different mechanisms are at play (Fig. 7*C*). As prior SAM binding has a positive allosteric effect on tRNA binding (vide supra, Table 2), it is conceivable that SAM binding may be necessary to induce a conformational change in TrmB, which structurally prepares TrmB for stable tRNA binding. As this conformational change has already occurred when TrmB is preincubated in the presence of SAM, the initial fluorescence decrease in the stopped-flow experiments likely reflects the bimolecular interaction of TrmB and tRNA. However, in the absence of SAM, it is likely that tRNA binding is rate-limited by a preceding unimolecular conformational change in TrmB which is slow due to the absence of SAM. Thus, the observed apparent rate is not dependent on the TrmB concentration. Thereby, our rapid kinetic stopped-flow experiments provide further insight into the role of SAM for facilitating tRNA binding to TrmB. Building on the mechanism of TrmB described here, it will be interesting to analyze in the future whether and how TrmB influences tRNA folding as it likely disrupts critical tertiary interactions in the tRNA elbow region. Thus, TrmB has the potential to also act as a tRNA chaperone as previously reported for *E. coli* TruB and TrmA (32, 50, 51).

### Structural features of tRNA binding by TrmB

In order to identify *E. coli* TrmB residues with roles in tRNA binding, we prepared four TrmB variants for characterization both by equilibrium filter-binding assays as well as our novel rapid kinetic stopped-flow assay. For the first time, this allows us to quantitatively investigate tRNA binding by TrmB independent of its catalytic activity allowing us to dissect the contribution of specific TrmB resides for these steps. Rapid kinetic stopped-flow experiments indicate that the D144A substitution may slightly impair the kinetics of tRNA association (Fig. 3). In comparison to TrmB D144A, the TrmB T217A variant has an approximately 3-fold lower affinity for tRNA as measured in equilibrium nitrocellulose-filtration binding experiments (Table 2). Interestingly, the rapid kinetic stopped-flow experiments provide further detailed insight into the mechanism of tRNA binding by TrmB variants. Here, we observed that tRNA associates to TrmB T217A much slower than to TrmB D144A (Fig. 6). Together, the equilibrium and rapid kinetic experiments thus demonstrate unambiguously that TrmB T217A is not only impaired in m^7^G46 formation as previously reported but that the substitution of T217 with alanine also impairs tRNA binding to TrmB. Similarly, even stronger defects with respect to the affinity and kinetics of tRNA binding were observed for TrmB R26A and TrmB R155A (Table 2 and Fig. 5). For both variants, the tRNA affinity in the presence of SAM is at least 5-fold decreased compared to TrmB D144A and the apparent association rate is reduced from approximately 60 s^−1^ to 2 s^−1^. As tRNA binding is severely compromised for the TrmB R26A and R155A variants, additional steps in tRNA association are observed in the rapid kinetic stopped-flow experiments which may reflect conformational events that are typically hidden. In summary, residues R26, T217, and R155 are critically involved in tRNA binding to TrmB. Interestingly, these residues map to three different areas on the surface of TrmB (Fig. 1*B*). As TrmB is a relatively small enzyme of only 27 kDa, our results suggest that TrmB utilizes its extended surface to interact with tRNA. Such a relatively large interaction site between a modification enzyme and tRNA is not uncommon and was also reported for the human pseudouridine synthase PUS7 (52). Further structural studies will be needed to uncover the precise orientation of tRNA relative to *E. coli* TrmB.

Most recently, two publications reported structures of the human TrmB homolog, METTL1 in complex with its partner protein, WDR4, and tRNA, in the presence and absence of SAM or SAH obtained by cryo-EM (47, 48). The biochemical results described here for *E. coli* TrmB corroborate several similar features and highlight important differences for tRNA binding by monomeric bacterial TrmB and its heterodimeric human counterpart METTL1. First, for both TrmB and METTL1, the entire positively charged surface of the relatively small methyltransferase is utilized for tRNA binding. However, the METTL1/WDR4 quaternary structures demonstrate for the first time that WDR4 also makes direct contacts with tRNA, thereby increasing the affinity of METTL1 for tRNA (47, 48). These interactions obviously cannot take place for monomeric TrmB and may provide an explanation why the affinity of monomeric TrmB for tRNA is comparatively weak to that of the METTL1-WDR4 heterodimer with affinities of ∼6 μM and ∼90 nM respectively (Fig. 5*A* and Table 2, (48)). Secondly, comparison of the METTL1⋅WDR4⋅tRNA structure to that of the METTL1⋅WDR4⋅tRNA⋅SAH structure demonstrates that although METTL1⋅WDR4 can form a specific complex with tRNA, several local METTL1 conformational changes take place upon cofactor (SAH) binding to facilitate stable METTL1 binding and tRNA methylation. Particularly, in the presence of cofactor, the METTL1 N terminus becomes ordered, sandwiching itself between the tRNA and cofactor. This supports the accession of the buried G46 nucleobase by METTL1 and facilitates the overall METTL1 movement nearer to the tRNA and protrusion of the catalytic loop toward that target base (48). Although the details of methyltransferase conformation change may be distinct between the heterodimeric human methyltransferase and monomeric *E. coli* TrmB, structural rearrangements upon cofactor binding for the METTL1⋅WDR4⋅tRNA provides precedent for the suggested local methyltransferase conformational changes upon SAM binding to TrmB facilitating stable tRNA binding, consistent with our equilibrium binding experiments with WT TrmB (Fig. 5 and Table 2) and our rapid kinetic analyses of inactive TrmB (Fig. 7). Finally, the new METTL1⋅WDR4⋅tRNA complexes reveal cofactor and tRNA binding are necessary for organization of the flexible METTL1 N terminus which coordinates cofactor binding with structural changes within METTL1, WDR4, and the tRNA (47, 48). Here, we show alanine substitution within the TrmB N terminus at R26 impairs tRNA binding (Figs. 5*D* and 6*C*; Table 2), suggesting an important role for this residue for the interaction between TrmB and tRNA. Although the R26 residue is not conserved in human METTL1 and is generally a glutamine (flanked by an arginine on each side) within eukaryotic TrmB homologs, our findings suggest a conserved role for arginine residues in the flexible N terminus among TrmB/METTL1 homologs.

## Conclusion

Taken together, our results show that TrmB is a biologically relevant enzyme that confers a fitness advantage to *E. coli* under oxidative stress. As previous reports suggest that this effect may be tRNA-specific, it is crucial to understand the interaction of TrmB with tRNA. As a first critical step in this direction, we established a new rapid kinetic assay complementing standard equilibrium binding assays such as nitrocellulose filtration. Thereby, we have dissected the molecular mechanism of TrmB. Our data suggest that binding of SAM to TrmB induces a conformational change that is required for fast and tight tRNA binding, which is corroborated by two recent METTL1⋅tRNA complex structures (47, 48), suggesting the potential for a common mechanism for homologous N7-methylguanosine methyltransferases with distinct quaternary structures. Moreover, we show three residues located at different areas on the TrmB surface (R26, T217, and R155) are critically contributing to the stable and fast binding of tRNA to this enzyme. Following tRNA binding, the formation of m^7^G46 is the rate-limiting step which is followed by rapid release of the modified tRNA (Fig. 7*C*). This insight into the molecular mechanism of TrmB thus lays the ground for future studies to address the role of TrmB-mediated modification of different tRNAs and for structural studies of the TrmB–tRNA complex.

## Experimental procedures

### Buffers and reagents

Experiments were performed in TAKEM_4_ buffer (50 mM Tris–HCl pH 7.5, 70 mM NH_4_Cl, 30 mM KCl, 1 mM EDTA, 4 mM MgCl_2_). [C5-^3^H]UTP for *in vitro* transcription of [^3^H]tRNA^Phe^ was purchased from Moravek Biochemicals. 5-IAF was from Sigma-Aldrich. Nonradioactive SAM was purchased from New England Biolabs and [methyl-^3^H]SAM was purchased from PerkinElmer. All other chemicals were purchased from Thermo Fisher Scientific.

### Hydrogen peroxide growth comparison for *ΔtrmB* and its parental strain

The identity of the *ΔtrmB* strain from the Keio collection was validated by colony PCRs targeting the region upstream (5′-GCTGCAACTTCCTCAAAGG-3′) and downstream (5′-CGTCACTGAAAGTGCTGCC-3′) of the *trmB* locus and the kanamycin cassette (k1/k2) (53). For growth analysis, four biological replicates for each the *ΔtrmB* strain and its parental strain, BW25113, were grown overnight in 3 to 5 ml LB in the presence of 50 μg/ml kanamycin (*ΔtrmB*) or in the absence of antibiotic (WT). Cells were resuspended in fresh LB medium lacking antibiotic and diluted to an A_600_ of 0.1 in 150 μl LB containing 5 mM H_2_O_2_. Cultures were incubated at 37 °C for 48 h with continuous shaking and the absorbance was measured every 15 min at 600 nm in an Eon BioTek 96-well plate reader. The average A_600_ reading of at least three biological replicates for each strain was plotted *versus* time with error bars representing the SEM.

### Site-directed mutagenesis to prepare TrmB variants

Expression plasmids for TrmB variant expression were prepared from the pET28a-TrmB plasmid (31) using Quikchange site-directed mutagenesis with the following overlapping primers:

TrmB D144A: 5′-TTTTCCCTGCCCCGTGGCACAAAGCGC-3′ and 5′- GCCACGGGGCAGGGAAAAAGAGCTGC-3′.

TrmB T217A: 5′- CCGGTGGCGAAATTTGAACAACGTGG-3′ and 5′-CAAATTTCGCCACCGGACGTGATGCC-3′.

TrmB R26A: 5′- TTTGTGCGCGCCCAGGGGCGACTGAC-3′ and 5′-CCCCTGGCGGCGCACAAAACTACGG-3′.

TrmB R155A: 5′- AATAAACGCGCTATCGTTCAGGTGCCG-3′ and 5′- GAACGATAGCGCGTTTATTATGGCGCGC-3′.

The sequences of pET28a-TrmB D144A, pET28a-TrmB R26A, pET28a-TrmB R155A, pET28a-TrmB T217A were confirmed by Sanger sequencing (Genewiz).

### Protein expression and purification

Plasmids encoding WT or variant TrmB were transformed into BL21 (DE3) cells. Similarly, plasmids pBH113 and pBH402 for ThiI and IscS overexpression were transformed into BL21 (DE3) for overexpression as described in (31). In brief, for overexpression, cells were grown at 37 °C in LB broth supplemented with 50 μg/ml kanamycin or 100 μg/ml ampicillin. Protein overexpression was induced at an A_600_ of approximately 0.6 with 1 mM IPTG. After 3 h, cells were collected by centrifugation at 5000*g* for 15 min, flash frozen, and stored at −80 °C.

Proteins were purified *via* their amino-terminal hexahistidine tag using nickel-sepharose followed by Superdex 75 (XK 26/100) chromatography as described (49). TrmB protein concentrations were determined by absorbance at 280 nm using an extinction coefficient of 27,960 M^−1^ cm^−1^ (calculated using ProtParam (54)). Similarly, ThiI and IscS concentrations were determined by A_280_ using experimentally determined extinction coefficients 63,100 and 25,400 M^−1^ cm^−1^, respectively (55). Protein concentrations were validated using comparative SDS-PAGE and Bradford assays.

### tRNA^Phe^ preparation

*E. coli* tRNA^Phe^ was prepared as described (49) by amplification from the pCF0 plasmid (56) followed by *in vitro* transcription. The resulting tRNA^Phe^ was purified by phenol extraction and Superdex 75 (XK 26/100) chromatography. For uniformly tritium-labeled tRNA^Phe^, [5-^3^H]UTP was included in the reaction and [5-^3^H]tRNA^Phe^ was purified by Nucleobond Xtra Midi anion exchange gravity columns (Machery-Nagel) (49).

### tRNA^Phe^ modification and fluorescent labeling

In order to introduce a specific fluorescent label at position 8 of the tRNA structure, purified ThiI and IscS enzymes were used to prepare s^4^U8-tRNA^Phe^. For this reaction, tRNA^Phe^ was first folded in TAKEM_4_ buffer by heating to 65 °C for 5 min and cooling to room temperature for at least 10 min. Folded tRNA^Phe^ at a final concentration of 5 μM was incubated with 3 μM IscS and 3 μM ThiI in the presence of 40 μM pyridoxal-5ʹ-phosphate, 4 mM ATP, 500 μM L-cysteine, 0.07 U/μl RiboLock RNase inhibitor (Thermo Fisher Scientific), and 1 mM dithiothreitol in TAKEM_4_ buffer for at least 2 h at 37 °C in a 20 ml total volume. The reaction was stopped by enzyme denaturation *via* heating at 80 °C for 15 min and enzymes were then removed by phenol/chloroform extraction. Following isopropanol precipitation to reduce the volume, reaction cofactors were removed by Superdex 75 (10/300 Gl) chromatography. tRNA thiolation was validated to be at least 80% by ImageJ analysis of 5 to 100 pmol of tRNA on a 20 μM [(N-acryloylamino)phenyl]mercuric chloride, 7 M urea, 10% polyacrylamide gel (57).

Fluorescent labeling of the s^4^U8 residue was achieved by incubating 60 μM s^4^U8-tRNA^Phe^ with 3.2 mM 5-IAF in 12 mM Hepes-KOH pH 8.2 containing 80% (v/v) dimethyl sulfoxide at 65 °C in the dark for 4 h, similar to (36). To remove unincorporated dye, at least eight successive phenol extractions were performed until the organic layer was no longer yellow. To remove phenol, two chloroform extractions were performed followed by ethanol precipitation. The final fluorescein-s^4^U8-tRNA^Phe^ was resuspended in water and the tRNA concentration was determined spectrophotometrically at 260 nm. The labeling efficiency was determined by absorbance at 492 nm and was typically ∼2 to 15%.

### Stopped-flow to monitor TrmB–tRNA^Phe^ binding

Following tRNA folding, fluorescein-s^4^U8-tRNA^Phe^ (final concentration: 1 μM) was rapidly mixed with TrmB at a final concentration between 3 to 20 μM in TAKEM_4_ buffer in a KinTek SF-2004 stopped-flow apparatus at 20 °C. Fluorescein was excited at 480 nm and emission monitored from 505 nm onward. Relative fluorescence (*Y*) was plotted against time (*t*) and traces were fit to a one-, two-, or three-exponential function to determine apparent rates (*k*_app_) using TableCurve 2D:

$$Y=Y_{\infty }+Amp\times \exp\;\left(-k_{app}\times t\right)$$

$$Y=Y_{\infty }+{Amp}_{1}\times \exp\left(-k_{app1}\times t\right)+{Amp}_{2}\times \exp\;\left(-k_{app2}\times t\right)$$

$$Y=Y_{\infty }+{Amp}_{1}\times \exp\left(-k_{app1}\times t\right)+{Amp}_{2}\times \exp\left(-k_{app2}\times t\right)+{Amp}_{3}\times \exp\left(-k_{app3}\times t\right)$$

Data shown are averages of at least eight independent time courses.

For TrmB D144A, apparent rates were plotted against enzyme concentration and fit with a linear equation to determine the association rate constant *k*_1_:

$$k_{app}=k_{1}\times \left[enzyme\right]+k_{-1}$$

### Nitrocellulose filtration to determine affinity of TrmB for tRNA

Prior to the reaction, [^3^H]tRNA^Phe^ was refolded in TAKEM_4_ buffer by heating and cooling as described above. Refolded [^3^H]tRNA^Phe^ (40 nM) was incubated with increasing concentrations of TrmB WT or variant enzyme (0–30 μM) in TAKEM_4_ buffer in the presence or absence of 50 μM SAM for 10 min at room temperature.

As described in (49), the enzyme–tRNA mixture was filtered through a nitrocellulose membrane under vacuum and the proportion of bound tRNA was determined by scintillation counting. To determine the dissociation constant (*K*_*D*_), percent tRNA bound (*Bound*) was plotted as a function of enzyme concentration ([*enzyme*]) and fit with a hyperbolic equation using GraphPad Prism software (https://www.graphpad.com/):

$$Bound={Bound}_{\max}\times \left[enzyme\right]/\left(K_{D}+\left[enzyme\right]\right)$$

### Methylation assay to monitor tRNA modification by TrmB

Refolded tRNA^Phe^ (1 μM) was incubated with TrmB (5 μM for single-turnover assays or 50 nM for multiple-turnover experiments) in the presence of 50 μM [^3^H]SAM (PerkinElmer; diluted with nonradioactive SAM to achieve a final specific activity of 800 dpm/pmol) in TAKEM_4_ buffer at the temperature indicated (37 °C for Fig. 4 and 20 °C for Fig. S1). Reaction samples (190 μl for reactions mixed in a KinTek Quench Flow or 20 μl for reactions not performed in quench flow) were quenched at indicated times by spotting onto Whatman filter papers presoaked with 5% (w/v) trichloroacetic acid. After letting filters dry for at least 10 min, filters were washed three times with 5% (w/v) trichloroacetic acid followed by a final wash in anhydrous ethanol. After drying for ∼30 min at 60 °C, the radioactivity remaining on filters was quantified by scintillation counting in 4 ml EcoLite (+) scintillation cocktail (MP Biomedicals). Percent methylation or raw decays per minute was plotted against time. Single-turnover methylation time courses were fit with a one-exponential equation to determine the apparent rate, *k*_*app*_:

$$Y=Y_{\infty }+Amp\times \exp\;\left(-k_{app}\times t\right)$$

## Data availability

All data are contained within the article.

## Supporting information

This article contains supporting information.

## Conflict of interest

The authors declare that they have no conflicts of interest with the contents of this article.

## References

[1] Boccaletto P., Machnicka M.A., Purta E., Piatkowski P., Baginski B., Wirecki T.K. (2018). MODOMICS: a database of RNA modification pathways. 2017 update. Nucleic Acids Res..

[2] Swinehart W.E., Jackman J.E. (2015). Diversity in mechanism and function of tRNA methyltransferases. RNA Biol..

[3] Hou Y.M., Perona J.J. (2010). Stereochemical mechanisms of tRNA methyltransferases. FEBS Lett..

[4] Tomikawa C. (2018). 7-Methylguanosine modifications in transfer RNA (tRNA). Int. J. Mol. Sci..

[5] Agris P.F., Sierzputowska-Gracz H., Smith C. (1986). Transfer RNA contains sites of localized positive charge: carbon NMR studies of [13C]methyl-enriched *Escherichia coli* and yeast tRNAPhe. Biochemistry.

[6] Hurd R.E., Reid B.R. (1979). Nuclear magnetic resonance studies on the tertiary folding of transfer ribonucleic acid: assignment of the 7-methylguanosine resonance. Biochemistry.

[7] De Bie L.G., Roovers M., Oudjama Y., Wattiez R., Tricot C., Stalon V. (2003). The yggH gene of Escherichia coli encodes a tRNA (m7G46) methyltransferase. J. Bacteriol..

[8] Zhou H., Liu Q., Yang W., Gao Y., Teng M., Niu L. (2009). Monomeric tRNA (m(7)G46) methyltransferase from Escherichia coli presents a novel structure at the function-essential insertion. Proteins.

[9] Cheng W., Gao A., Lin H., Zhang W. (2022). Novel roles of METTL1/WDR4 in tumor via m(7)G methylation. Mol. Ther. Oncolytics.

[10] Chen Y., Lin H., Miao L., He J. (2022). Role of N7-methylguanosine (m(7)G) in cancer. Trends Cell Biol..

[11] Orellana E.A., Liu Q., Yankova E., Pirouz M., De Braekeleer E., Zhang W. (2021). METTL1-mediated m(7)G modification of Arg-TCT tRNA drives oncogenic transformation. Mol. Cell.

[12] Ma J., Han H., Huang Y., Yang C., Zheng S., Cai T. (2021). METTL1/WDR4-mediated m(7)G tRNA modifications and m(7)G codon usage promote mRNA translation and lung cancer progression. Mol. Ther..

[13] Dai Z., Liu H., Liao J., Huang C., Ren X., Zhu W. (2021). N(7)-Methylguanosine tRNA modification enhances oncogenic mRNA translation and promotes intrahepatic cholangiocarcinoma progression. Mol. Cell.

[14] Shaheen R., Abdel-Salam G.M., Guy M.P., Alomar R., Abdel-Hamid M.S., Afifi H.H. (2015). Mutation in WDR4 impairs tRNA m(7)G46 methylation and causes a distinct form of microcephalic primordial dwarfism. Genome Biol..

[15] Alexandrov A., Grayhack E.J., Phizicky E.M. (2005). tRNA m7G methyltransferase Trm8p/Trm82p: evidence linking activity to a growth phenotype and implicating Trm82p in maintaining levels of active Trm8p. RNA.

[16] Alexandrov A., Chernyakov I., Gu W., Hiley S.L., Hughes T.R., Grayhack E.J. (2006). Rapid tRNA decay can result from lack of nonessential modifications. Mol. Cell.

[17] Thongdee N., Jaroensuk J., Atichartpongkul S., Chittrakanwong J., Chooyoung K., Srimahaeak T. (2019). TrmB, a tRNA m7G46 methyltransferase, plays a role in hydrogen peroxide resistance and positively modulates the translation of katA and katB mRNAs in Pseudomonas aeruginosa. Nucleic Acids Res..

[18] Takano Y., Takayanagi N., Hori H., Ikeuchi Y., Suzuki T., Kimura A. (2006). A gene involved in modifying transfer RNA is required for fungal pathogenicity and stress tolerance of Colletotrichum lagenarium. Mol. Microbiol..

[19] Tomikawa C., Yokogawa T., Kanai T., Hori H. (2010). N7-Methylguanine at position 46 (m7G46) in tRNA from Thermus thermophilus is required for cell viability at high temperatures through a tRNA modification network. Nucleic Acids Res..

[20] Zegers I., Gigot D., van Vliet F., Tricot C., Aymerich S., Bujnicki J.M. (2006). Crystal structure of Bacillus subtilis TrmB, the tRNA (m7G46) methyltransferase. Nucleic Acids Res..

[21] Tomikawa C., Ochi A., Hori H. (2008). The C-terminal region of thermophilic tRNA (m7G46) methyltransferase (TrmB) stabilizes the dimer structure and enhances fidelity of methylation. Proteins.

[22] Alexandrov A., Martzen M.R., Phizicky E.M. (2002). Two proteins that form a complex are required for 7-methylguanosine modification of yeast tRNA. RNA.

[23] Leulliot N., Chaillet M., Durand D., Ulryck N., Blondeau K., van Tilbeurgh H. (2008). Structure of the yeast tRNA m7G methylation complex. Structure.

[24] Liu Q., Gao Y., Yang W., Zhou H., Gao Y., Zhang X. (2008). Crystallization and preliminary crystallographic analysis of tRNA (m(7)G46) methyltransferase from Escherichia coli. Acta Crystallogr. Sect. F Struct. Biol. Cryst. Commun..

[25] Blersch K.F., Burchert J.P., August S.C., Welp L., Neumann P., Köster S. (2021). Structural model of the M7G46 methyltransferase TrmB in complex with tRNA. RNA Biol..

[26] Purta E., van Vliet F., Tricot C., De Bie L.G., Feder M., Skowronek K. (2005). Sequence-structure-function relationships of a tRNA (m7G46) methyltransferase studied by homology modeling and site-directed mutagenesis. Proteins.

[27] Tomikawa C., Takai K., Hori H. (2018). Kinetic characterization of substrate-binding sites of thermostable tRNA methyltransferase (TrmB). J. Biochem..

[28] Bimai O., Arragain S., Golinelli-Pimpaneau B. (2020). Structure-based mechanistic insights into catalysis by tRNA thiolation enzymes. Curr. Opin. Struct. Biol..

[29] He N., Zhou J., Bimai O., Oltmanns J., Ravanat J.L., Velours C. (2022). A subclass of archaeal U8-tRNA sulfurases requires a [4Fe-4S] cluster for catalysis. Nucleic Acids Res..

[30] Johnson A.E., Adkins H.J., Matthews E.A., Cantor C.R. (1982). Distance moved by transfer RNA during translocation from the A site to the P site on the ribosome. J. Mol. Biol..

[31] Schultz S.K., Kothe U. (2020). tRNA elbow modifications affect the tRNA pseudouridine synthase TruB and the methyltransferase TrmA. RNA.

[32] Keffer-Wilkes L.C., Soon E.F., Kothe U. (2020). The methyltransferase TrmA facilitates tRNA folding through interaction with its RNA-binding domain. Nucleic Acids Res..

[33] Meyer B., Immer C., Kaiser S., Sharma S., Yang J., Watzinger P. (2020). Identification of the 3-amino-3-carboxypropyl (acp) transferase enzyme responsible for acp3U formation at position 47 in Escherichia coli tRNAs. Nucleic Acids Res..

[34] Fasnacht M., Gallo S., Sharma P., Himmelstoß M., Limbach P.A., Willi J. (2022). Dynamic 23S rRNA modification ho5C2501 benefits Escherichia coli under oxidative stress. Nucleic Acids Res..

[35] Chen Z., Zhu W., Zhu S., Sun K., Liao J., Liu H. (2021). METTL1 promotes hepatocarcinogenesis via m(7) G tRNA modification-dependent translation control. Clin. Transl Med..

[36] Milon P., Konevega A.L., Peske F., Fabbretti A., Gualerzi C.O., Rodnina M.V. (2007). Transient kinetics, fluorescence, and FRET in studies of initiation of translation in bacteria. Methods Enzymol..

[37] Betteridge T., Liu H., Gamper H., Kirillov S., Cooperman B.S., Hou Y.M. (2007). Fluorescent labeling of tRNAs for dynamics experiments. RNA.

[38] Liu C., Betteridge T., Hou Y.M. (2009). Fluorophore labeling to monitor tRNA dynamics. Methods Enzymol..

[39] Barraud P., Gato A., Heiss M., Catala M., Kellner S., Tisné C. (2019). Time-resolved NMR monitoring of tRNA maturation. Nat. Commun..

[40] Heiss M., Hagelskamp F., Marchand V., Motorin Y., Kellner S. (2021). Cell culture NAIL-MS allows insight into human tRNA and rRNA modification dynamics *in vivo*. Nat. Commun..

[41] Lauhon C.T., Erwin W.M., Ton G.N. (2004). Substrate specificity for 4-thiouridine modification in Escherichia coli. J. Biol. Chem..

[42] Schultz S.K., Kothe U. (2021). Partially modified tRNAs for the study of tRNA maturation and function. Methods Enzymol..

[43] Matsumoto K., Toyooka T., Tomikawa C., Ochi A., Takano Y., Takayanagi N. (2007). RNA recognition mechanism of eukaryote tRNA (m7G46) methyltransferase (Trm8-Trm82 complex). FEBS Lett..

[44] Okamoto H., Watanabe K., Ikeuchi Y., Suzuki T., Endo Y., Hori H. (2004). Substrate tRNA recognition mechanism of tRNA (m7G46) methyltransferase from Aquifex aeolicus. J. Biol. Chem..

[45] Guy M.P., Phizicky E.M. (2014). Two-subunit enzymes involved in eukaryotic post-transcriptional tRNA modification. RNA Biol..

[46] Graille M. (2022). Division of labor in epitranscriptomics: what have we learnt from the structures of eukaryotic and viral multimeric RNA methyltransferases?. Wiley Interdiscip. Rev. RNA.

[47] Li J., Wang L., Hahn Q., Nowak R.P., Viennet T., Orellana E.A. (2023). Structural basis of regulated m(7)G tRNA modification by METTL1-WDR4. Nature.

[48] Ruiz-Arroyo V.M., Raj R., Babu K., Onolbaatar O., Roberts P.H., Nam Y. (2023). Structures and mechanisms of tRNA methylation by METTL1-WDR4. Nature.

[49] Wright J.R., Keffer-Wilkes L.C., Dobing S.R., Kothe U. (2011). Pre-steady-state kinetic analysis of the three Escherichia coli pseudouridine synthases TruB, TruA, and RluA reveals uniformly slow catalysis. RNA.

[50] Keffer-Wilkes L.C., Veerareddygari G.R., Kothe U. (2016). RNA modification enzyme TruB is a tRNA chaperone. Proc. Natl. Acad. Sci. U. S. A..

[51] Porat J., Kothe U., Bayfield M.A. (2021). Revisiting tRNA chaperones: new players in an ancient game. RNA.

[52] Guegueniat J., Halabelian L., Zeng H., Dong A., Li Y., Wu H. (2021). The human pseudouridine synthase PUS7 recognizes RNA with an extended multi-domain binding surface. Nucleic Acids Res..

[53] Baba T., Ara T., Hasegawa M., Takai Y., Okumura Y., Baba M. (2006). Construction of *Escherichia coli* K-12 in-frame, single-gene knockout mutants: The Keio collection. Mol. Syst. Biol..

[54] Gill S.C., von Hippel P.H. (1989). Calculation of protein extinction coefficients from amino acid sequence data. Anal. Biochem..

[55] Mueller E.G., Palenchar P.M., Buck C.J. (2001). The role of the cysteine residues of ThiI in the generation of 4-thiouridine in tRNA. J. Biol. Chem..

[56] Sampson J.R., DiRenzo A.B., Behlen L.S., Uhlenbeck O.C. (1989). Nucleotides in yeast tRNAPhe required for the specific recognition by its cognate synthetase. Science.

[57] Igloi G.L. (1988). Interaction of tRNAs and of phosphorothioate-substituted nucleic acids with an organomercurial. Probing the chemical environment of thiolated residues by affinity electrophoresis. Biochemistry.

[58] Byrne R.T., Konevega A.L., Rodnina M.V., Antson A.A. (2010). The crystal structure of unmodified tRNAPhe from Escherichia coli. Nucleic Acids Res..

