# Supplementary material for: Molecular mechanism of tRNA binding by the Escherichia coli N7 guanosine methyltransferase TrmB
Source: J Biol Chem. 2023 Mar 16;299(5):104612. doi: 10.1016/j.jbc.2023.104612 (PMC10130221; doi:10.1016/j.jbc.2023.104612)
Supplement: Supplemental Table S1 and Figure S1 [file mmc1.pdf]

**Supporting Information**

# Molecular mechanism of tRNA binding by the *Escherichia coli* N7 guanosine methyltransferase TrmB

Sarah K. Schultz [1,2], Kieran Meadows [1], and Ute Kothe [1,2*]

[1] Alberta RNA Research and Training Institute (ARRTI), Department of Chemistry and Biochemistry, University of Lethbridge, Lethbridge, Alberta, Canada

[2] Department of Chemistry, University of Manitoba, Winnipeg, Manitoba, Canada

* To whom correspondence should be addressed: ute.kothe@umanitoba.ca

**Table S1. Average apparent rates ($k_{app}$) for the association of TrmB variants and fluorescein-s$^4$U8-tRNA$^{Phe}$.** Rates were determined by fitting data shown in Figure 6 with 1-, 2-, or 3-exponential equations. $k_{app}$ increase refers to an event wherein fluorescence is increased, whereas $k_{app1}$ and $k_{app2}$ correspond to decreases in fluorescence.

| | **$k_{app}$ increase ($s^{-1}$)** | **$k_{app1}$ ($s^{-1}$)** | **$k_{app2}$ ($s^{-1}$)** |
|---|---|---|---|
| TrmB D144A | N/A | 62 ± 5 | N/A |
| TrmB T217A | N/A | 8 ± 1 | 0.2 ± 0.02 |
| TrmB R26A | N/A | 2 ± 0.2 | 0.04 ± 0.002 |
| TrmB R155A | 176 ± 46 | 2 ± 1 | 0.03 ± 0.002 |

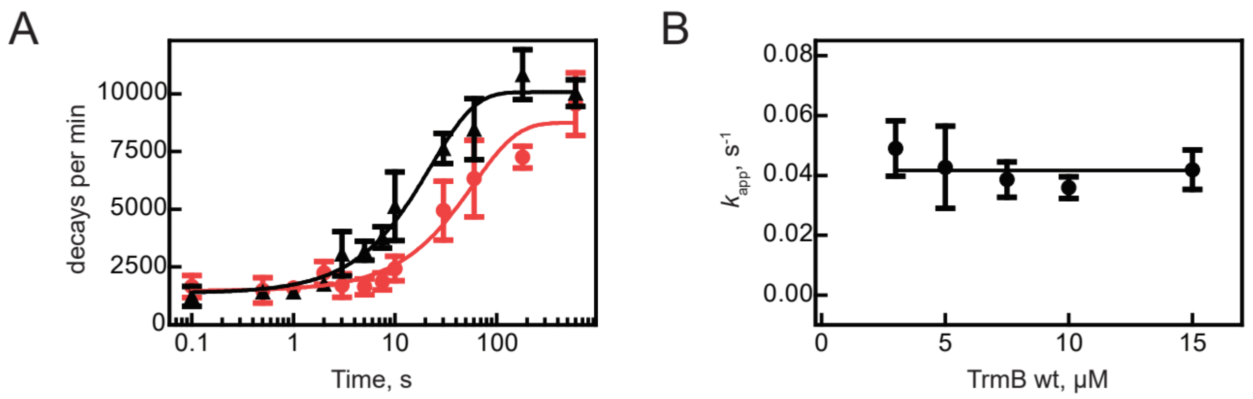


**Figure S1. Rapid tRNA methylation by TrmB at 20°C. (A)** Time courses of tRNA methylation are recorded using a quench-flow apparatus. TrmB (5 µM) in the presence of 50 µM SAM is rapidly mixed with 1 µM of unmodified *in vitro* transcribed $tRNA^{Phe}$ (black triangles) or fluorescein-$s^4U8$-$tRNA^{Phe}$ (red circles) at 20°C. Error bars display the average of three replicates. Both time courses were fit with a 1-exponential equation to determine the apparent rate of methylation ($k_{app}$), which was determined to be 0.04 ± 0.01 $s^{-1}$ for unmodified $tRNA^{Phe}$ and 0.02 ± 0.01 $s^{-1}$ for fluorescein-$s^4U8$-$tRNA^{Phe}$. **(B)** Concentration dependence of tRNA methylation by TrmB. Time courses of unmodified tRNA methylation by TrmB were fit with 1-exponential equations, and the resulting $k_{app}$ was plotted against TrmB concentration. Apparent rates are not to be dependent on TrmB concentration, and the horizontal line represents an average $k_{app}$ of 0.04 ± 0.01 $s^{-1}$.
